# Thermodynamic Description of Dilution and Dissolution Processes in the MgCl_2_−CsCl−H_2_O Ternary System

**DOI:** 10.3390/ma14144047

**Published:** 2021-07-20

**Authors:** Valeriia Baranauskaite, Maria Belysheva, Olga Pestova, Yuri Anufrikov, Mikhail Skripkin, Yuri Kondratiev, Vassily Khripun

**Affiliations:** 1 Department of Chemistry, Natural Science Faculty, Ben Gurion University of Negev, Beer Sheva 84105, Israel; valeriiebar@gmail.com; 2 Chemistry Institute, Saint Petersburg State University, 198504 Saint Petersburg, Russia; o.pestova@spbu.ru (O.P.); m.skripkin@spbu.ru (M.S.); y.kondratiev@spbu.ru (Y.K.); v.khripun@spbu.ru (V.K.); 3 Research Park, Center for Thermogravimetric and Calorimetric Research, Saint Petersburg State University, 198504 Saint Petersburg, Russia; y.anufrikov@spbu.ru

**Keywords:** heats of dilution, heats of dissolution, double salts, carnallite like salts, relative partial molar properties, calorimetry, X-ray crystallography

## Abstract

Thermodynamic data on the properties of the water-based electrolyte systems are very valuable for fundamental physical chemistry and for industrial applications. The missing data both on the dilution and dissolution enthalpies for the ternary CsCl−MgCl_2_−H_2_O mixed electrolyte system were investigated by means of the calorimetry method. The dilution calorimetry was performed at 298 K for the set of solutions from diluted to concentrated at constant ratio Cs^+^/Mg^2+^ =1.8. The relative partial molar enthalpies, ideal, total, and excess ones were calculated. By means of the dissolution calorimetry, the standard enthalpies of formation, the enthalpies, and entropies for the double salt formation from simple salts were evaluated. The results obtained indicate that entropy as the major factor affecting the formation of the joint compound, both in the liquid and solid phases. These data can be implemented in thermodynamic databases and allow for accurate thermodynamic calculations for the salts extraction from natural water sources and for its possible application as thermochemical energy storage.

## 1. Introduction

With the fast development of the theoretical and calculation methods, the need of the fundamental properties of the compounds arises. The thermodynamic properties of the compounds and systems are among those key parameters which undoubtedly play a crucial role in the physical chemistry field. Application of thermodynamic functions go far beyond theoretical calculations. It is essential for chemical reactions’ heat effect and direction calculations, for the multicomponent complex system phase diagram predictions. It is used to estimate the equilibrium conditions of the relevant system, etc. Particularly, the thermodynamical modeling is extremely useful for the prediction of properties of multicomponent systems. It is useful for systems imitating the composition of the natural water reservoirs, for the extraction of evaporites from the waters and other sources. Sometimes, it is the only way to receive pure and cheap enough chemicals. Among other ore minerals, carnallite is a natural source of the MgCl_2_ [1], KCl [2], and includes insignificant impurities of CsCl and RbCl [3]. However, carnallite and carnallite-like minerals of the MCl·MgCl_2_·nH_2_O composition are interesting not only as natural sources of chemical compounds, but also they can be made use of in renewable thermochemical energy storage since their hydration reactions are exothermic [4,5,6]. With the intention to use carnallite alike compounds as inorganic materials such as analogues of the thermochemical energy storages, we investigated their thermodynamic properties and formation processes.

The comprehensive thermodynamical studies of carnallite alike compounds will help to expand the opportunities horizon for utilization of this type of minerals. The thermodynamical properties of the MgCl_2_−CsCl−H_2_O system were studied by different methods. The earlier studies are related to the solid MgCl_2_·CsCl·6H_2_O structure [7,8,9,10], whereas the more recent papers are focusing on the liquid phase of this ternary system [11,12,13]. However, no data describing enthalpies, which are needed for the complete thermodynamic description of this system, were found in the literature. The heat effect of transition process from ions in the solution to the solid phase can be described with thermodynamic functions in particular standard enthalpy of the formation of the complex salts; furthermore, it can be compared to the similar data of the akin inorganic salts in terms of the differences in their crystal structures. Formation of the solid phase in essence is a complex and sophisticated process which requires the description by means of a model. The chosen model is validated by the agreement between the conclusions about the thermodynamical properties of the system under study. Thereby, in this paper, we are presenting the experimentally obtained dilution heat effects of the water based carnallite-like MgCl_2_−CsCl−H_2_O system, the dissolution heat effects of the solid double salt MgCl_2_·CsCl·6H_2_O, and of the mixture of constituent simple salts CsCl and MgCl_2_.

## 2. Materials and Methods

### 2.1. Samples’ Preparation Details

All chemicals used in this work were of analytical grade. The distilled water was used in each experiment. In the current study, a set of ten solutions was prepared in the CsCl:MgCl_2_ concentration ratio 1.8. Here, the concentration range of CsCl is 3.73–0.13 M and corresponding MgCl_2_ is 2.07–0.07 M. The saturated solution of MgCl_2_ was prepared, and the concentration of the Mg^2+^ cations was determined using a complexometry titration method, and the concentration of Cl^-^ anions was determined by a Mohr method [14]. The comparison of these two methods showed a good agreement. The calculated volume of MgCl_2_ solution and the calculated mass of CsCl were added into the volumetric flask and diluted with distilled water to the mark. The solutions with smaller concentration were prepared from the stock solution by the dilution method.

The double salt MgCl_2_·CsCl·H_2_O was prepared from the most concentrated solution by slow evaporation in the dissicator under low pressure over anhydrous granular CaCl_2_. The obtained crystals were placed in the thin-walled glass tubes and vacuumed at 323 K for several hours.

### 2.2. X-ray Crystallography and Powder Diffraction

X-ray crystallography (XRC) analysis was performed on a single-crystal diffractometer Oxford Diffraction “Xcalibur” (Agilent Technologies, Santa Clara, CA, USA) The sample was frozen to 100 K. Results of X-ray analysis were obtained using the program Olex2. The structure was determined making use of the ShelXL program with the least squares method. Samples for powder diffraction were radiographed to determine the phase composition with automatic diffractometer Bruker Phaser D2 (Bruker, Billerica, MA, USA). The following parameters were used for the studies: radiation 1.5444 Å, tube operation mode 30 kV/10 mA, position sensitive detector, reflection geometry, Bragg–Brentano focusing scheme, sample rotation speed of 20 revolutions per minute, diffraction angle interval 2θ = 6–90${}^{\circ }$, T $=25{\;}^{\circ }$C, and the atmosphere is air. The sample was prepared by dry pressing the test mixture into a cuvette.

### 2.3. Dissolution Calorimetry

A calorimetric study was performed to determine the dissolution enthalpies of double salts and dry mixtures of components. A conductive microcalorimeter of the Calvet type (Etalon, Almaty, Kazakhstan), equipped with two differential calorimetric cells, was used in this work. This device recorded time-resolved difference in heat flow in the working cell and in the comparison cell. The detailed description of the equipment can be found elsewhere [15]. The calorimeter was calibrated with the well-known data for KCl dissolution enthalpy [16]. The exact mass of the KCl was weighed on the balances with error ±0.0005 g. In order to receive a good signal-to-noise ratio, the mass of the samples was taken at about 0.03 g and the water-to-salt ratio was around 6500. The exact mass, heat effect, and calorimeter constant calculation are given in the Table A1. The dissolution thermal effect of KCl was recorded for the set of 8 samples, and the calorimeter constant was calculated. Right before the calorimetric experiment, the samples were dried under heating in the vacuum for 7 hours and sealed in the tiny glass vials. A part from these vials (4 vials) was used for calorimetric experiment and another part (4 vials) was investigated with the powder diffraction method. The glass vials were carefully weighed before and after drying to obtain the weight of the dried samples. The 50 ml of distilled water were poured into the reference cell and into the working cell, and a sealed glass vial with a known amount of salt or mixture was placed inside the working cell. The molar ratio of salts under study to water was kept around 1:35,000, which corresponds to the infinitely diluted solution. After several hours when the baseline was established, the glass vial was broken, and the sample was dissolved. The heat effect was later integrated and the enthalpy of dissolution ($\Delta H_{sol}$) of the salts was calculated with the help of calorimeter constant and the corresponding integral value. The exact experimental $\Delta H_{sol}$ are given in the Table A2 for double salt MgCl_2_·CsCl·6H_2_O and Table A3 for mixture of MgCl_2_·6H_2_O and CsCl.

### 2.4. Dilution Calorimetry

The heats of dilution of the salts were measured using the TA Instruments Nano ITC 2G titration microcalorimeter (TA Instruments, New Castle, DE, USA). The volume of 1 ml of the solution under study was placed in the cylindrical cell of the calorimeter and equilibrated. The temperature was kept constant within ±0.0003 K at 298.15 K, and the stirring was constant at 350 rpm. After the equilibrium was achieved, distilled water was added as a set of 6 sequential injections each of 0.28 μL volume. The calorimetrical signals in forms of peaks were integrated and recalculated into the enthalpy of dilution. The defined heat effects were averaged and attributed to the differential molar enthalpies of dilution and the error of the measured values was no more than 4%. The amount of the added portions of water was rather small compared to the solution and the mole ratio was approximately 1:1700 depending on the solution concentration. This ratio corresponds to the addition of an infinitely small portion to an infinitely large solution. Thus, the defined heat effect equals partial molar enthalpy of dilution $\partial \Delta H_{m}/\partial m$.

## 3. Results and Discussion

### 3.1. X-ray Crystallography and Powder Diffraction

**Figure 1:**
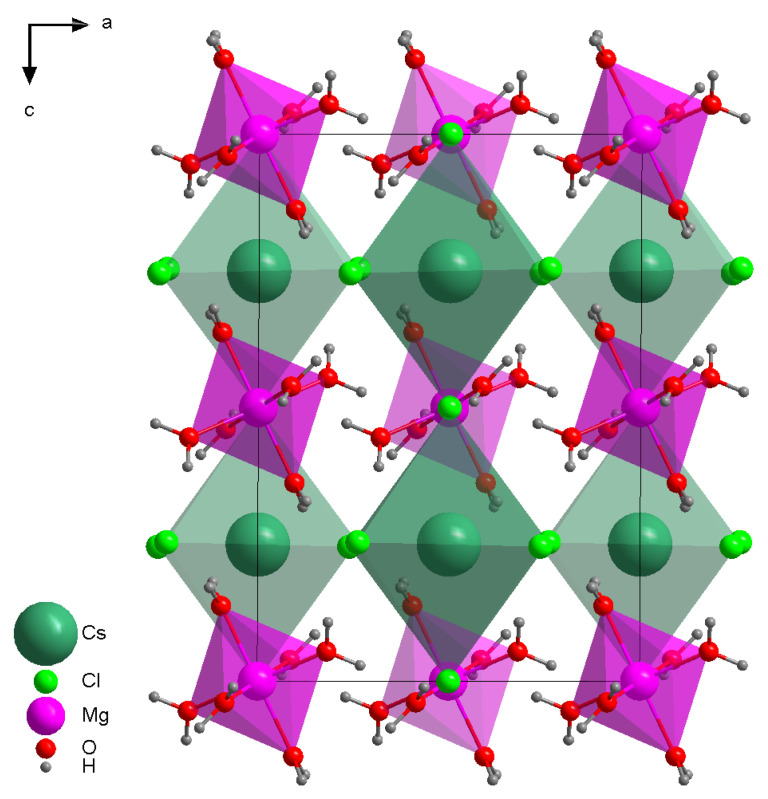
The unit cell of MgCl_2_·CsCl·6H_2_O double salt, ac projection.

**Figure 2:**
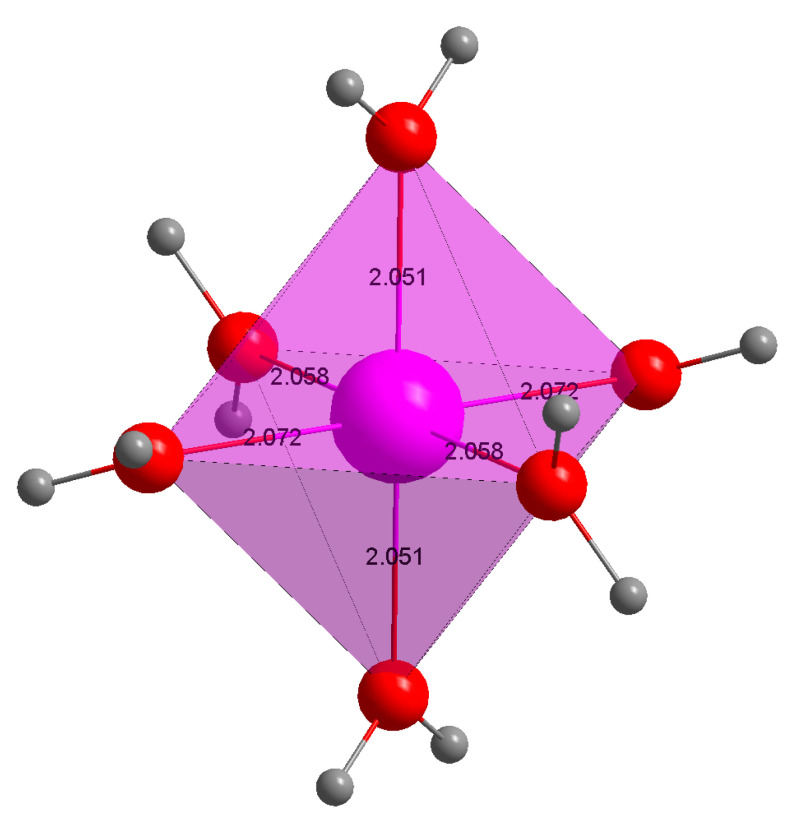
Octahedral environment of magnesium.

The crystal structure of the double salt MgCl_2_·CsCl·6H_2_O is well-known from the literature [8,17]. The former data are in very good agreement with parameters obtained from the crystal in our experiment. The unit cell of the double salt MgCl_2_·CsCl·6H_2_O is presented in Figure 1, and its principal crystallographic parameters are given in the Table A4. The Mg^2+^ ion is surrounded by six water molecules forming an isolated octahedra (Figure 2), which line up interspersed with rows of cesium rectangular prisms formed by chlorine. It should be noted that cesium polyhedra have common chlorine atoms with each neighboring cesium polyhedron; together, they form an extended layered structure. The distance between the nearest Mg atoms is 6.7258 Å, and the distances between O and Mg atoms in the octahedron vary from 2.051 Å to 2.071 Å. The distance between the nearest Cs atoms is 6.7275 Å, and the distances Cs−Cl are 3.3686 Å.

**Figure 3:**
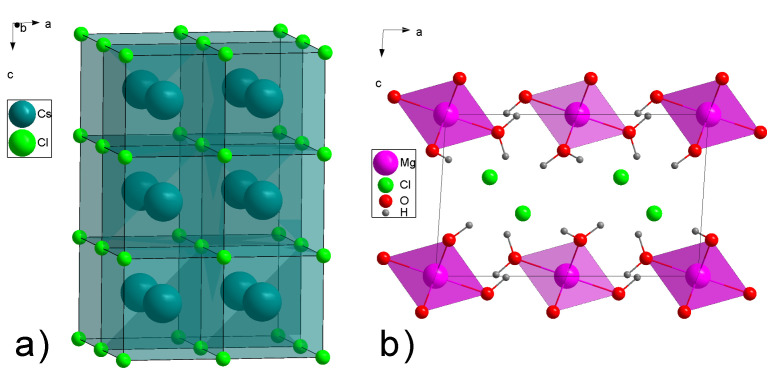
The structures of simple constituents salts. (**a**) the structure of anhydrous CsCl salt; (**b**) the structure of MgCl_2_·6H_2_O salt.

The double salt MgCl_2_·CsCl·6H_2_O is formed from two simple salts CsCl and MgCl_2_·6H_2_O. They can be obtained as individual compounds, and it is possible to follow the structural changes between simple constituent salts and double salt. The comprehensive representation of anhydrous CsCl structure is given in Figure 3 [18]. The major differences between simple CsCl and double salt lie in the symmetry of CsCl_8_ polyhedron. Whereas in simple salt it is clearly cubic with common chlorines shared through faces, in the double salt, it is octahedric with chlorines on the vertices. The distances between atoms in simple and double salts are also different as shown in Table 1. In the anhydrous CsCl salt, the distances between the nearest Cs are shrunk compared to the double salt, and the Cs−Cl distances are increased compared to the double salt.

The MgCl_2_−H_2_O system exhibits a huge variety of crystalline hydrates MgCl_2_·nH_2_O. In the double MgCl_2_·CsCl·6H_2_O salt, the nearest surroundings of Mg consist of six water molecules (Figure 2) that matches the Mg environment in simple MgCl_2_·6H_2_O crystalline hydrate [19,20]. The graphical representation of this salt is given in Figure 3. As it was mentioned, the magnesium surrounding in MgCl_2_·6H_2_O and MgCl_2_·CsCl·6H_2_O remains constant. The central Mg atom is surrounded by six water molecules and forms an octahedron that is isolated from the similar octahedra. The only changes in the simple salt compared to the double salt are the decreased distances between neighboring central Mg atoms in MgCl_2_·6H_2_O. As far as the distances between central Mg and surrounding water molecules are connected, they almost did not change as it is given in Table 2.

To conclude, we can tell that the formation mechanism of the double salt is a structurally forced intrusion according to the classification given in [21]. Both MgCl_2_ and CsCl have to change sufficiently their crystal structure in order to form the joint compound. The changes of the order and the structure obviously would influence the thermodynamical properties of the compound.

**Figure 4:**
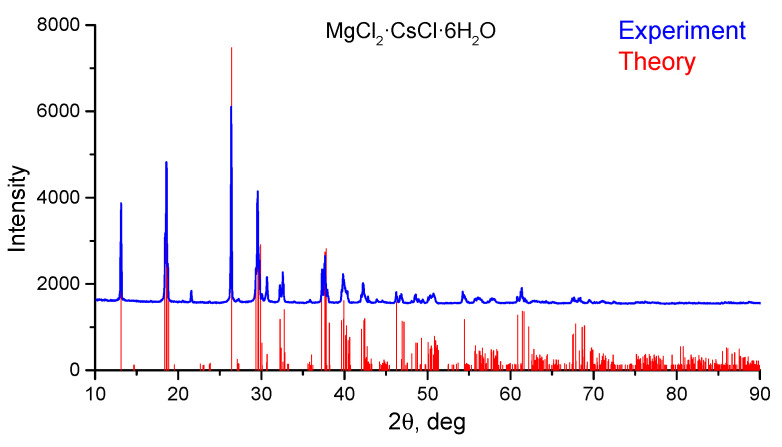
Comparison of calculated and experimental diffraction patterns of MgCl_2_·CsCl·6H_2_O double salt.

X-ray phase analysis was carried out in parallel with the calorimetric experiment for the same probes of MgCl_2_·CsCl·6H_2_O double salt. The thermal effects obtained from calorimetric measurements are extremely sensitive to impurities and excess of water. It was necessary to make sure that the samples contain only one phase with no impurities. The theoretical powder pattern for comparison with experimental one was produced using the program Diamond on the basis of the XRC experimental data. Comparison of the calculated and experimental diagrams of MgCl_2_·CsCl·6H_2_O crystal structure gives a good match as shown in Figure 4. It confirms the purity of the samples used in dissolution calorimetric experiments.

### 3.2. Dissolution Calorimetry

The dissolution enthalpies $\Delta H_{sol}$ of MgCl_2_·CsCl·6H_2_O double salt and mixture of constituent MgCl_2_·6H_2_O and CsCl salts were determined with the help of dissolution calorimetry. The experimentally obtained enthalpies are −111±4 kJ/mol for the double salt and −17.25±0.66 kJ/mol for mixture of salts. The detailed experimental results are given in Table A2 and Table A3, respectively. In its turn, the dissolution enthalpy of natural carnallite KCl·MgCl_2_·6H_2_O is known to be 9.34–10.8 kJ/mol [22,23]. Such a discrepancy between MgCl_2_·KCl·6H_2_O and MgCl_2_·CsCl·6H_2_O can be explained by the high stability of natural carnallite crystal structure. As given in the literature [8,24], the tolerance factor of natural carnallite is 1.061 and the octahedral factor is 0.73, whereas the tolerance factor of MgCl_2_·CsCl·6H_2_O is 0.963, and the octahedral factor is 0.91. Both carnallites lie in the area of the pervoskite type structure existence [25]; however, cesium carnallite is located very near to the boundaries, thereby it has less stable structure.

**Figure 5:**
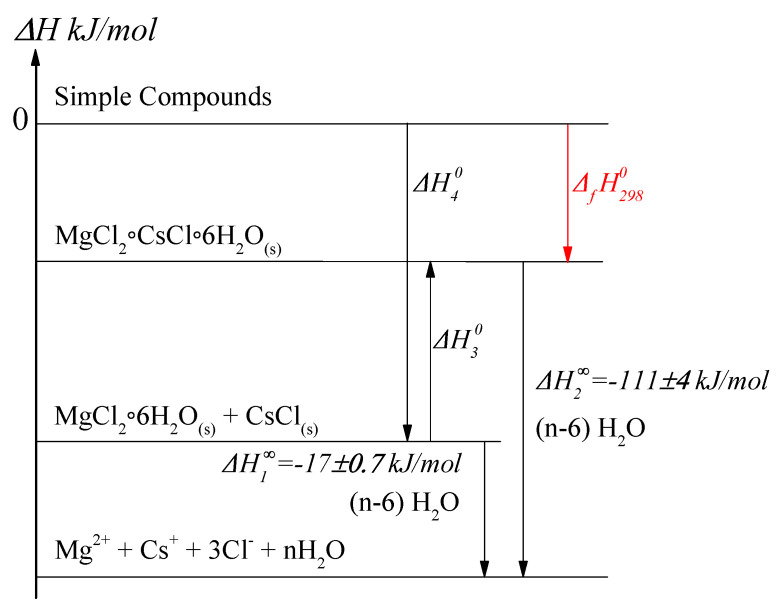
Thermochemical cycle for MgCl_2_·CsCl·6H_2_O dissolution.

In order to calculate the desired $\Delta_{f}H_{298}^{0}$, one needs to apply Hess’s law and build thermodynamic cycle (Figure 5), where for both cases the final states are infinitely diluted solutions.

Here:

- $\Delta H_{1}^{\infty }$ is dissolution enthalpy of salt mixture −17±0.66 kJ/mol
- $\Delta H_{2}^{\infty }$ is double salt dissolution enthalpy −111±4 kJ/mol
- $\Delta H_{3}^{0}$ is enthalpy of [MgCl_2_·6H_2_O]*s*+ [CsCl]*s* = [MgCl_2_·CsCl·6H_2_O]*s* reaction
- $\Delta H_{4}^{0}$ is the sum of the standard formation enthalpies of [MgCl_2_·6H_2_O]*s* and [CsCl]*s* salts
- $\Delta_{f}H_{298}^{0}$ is the standard formation enthalpy of the solid double salt

The formation of the double salt from constituent salts can be described by the reaction:

(1) $${\left[{\mathrm{MgCl}}_{2}\cdot 6H_{2}O\right]}_{s}+{\left[\mathrm{CsCl}\right]}_{s}={\left[{\mathrm{MgCl}}_{2}\cdot \mathrm{CsCl}\cdot 6H_{2}O\right]}_{s}.$$

The thermal effect of this reaction can be calculated according to the following formula:

(2) $$\Delta H_{3}^{0}=\Delta H_{1}^{\infty }-\Delta H_{2}^{\infty }=94\pm 4\mathrm{kJ}/\mathrm{mol}.$$

The $\Delta_{f}H_{298}^{0}\left(\begin{array}{c} {\mathrm{MgCl}}_{2}\cdot 6H_{2}O \end{array}\right)=-2498.852\pm 0.836$ kJ/mol and $\Delta_{f}H_{298}^{0}\left(\begin{array}{c} \mathrm{CsCl} \end{array}\right)=$ −442.437±0.251 kJ/mol were taken from the literature data [26,27,28] to calculate the $\Delta H_{4}^{0}$:

(3) $$\Delta H_{4}^{0}=\Delta_{f}H_{298}^{0}\left({\mathrm{MgCl}}_{2}\cdot 6H_{2}O\right)+\Delta_{f}H_{298}^{0}\left(\mathrm{CsCl}\right)=-2941.29\mathrm{kJ}/\mathrm{mol}.$$

Finally, the standard formation enthalpy of double salt $\Delta_{f}H_{298}^{0}\left(\begin{array}{c} {\mathrm{MgCl}}_{2}\cdot \mathrm{CsCl}\cdot 6H_{2}O \end{array}\right)$ is calculated according to the equation:

(4) $$\begin{array}{c} \Delta_{f}H_{298}^{0}=\Delta H_{4}^{0}+\Delta H_{3}^{0}=-2847\pm 4\mathrm{kJ}/\mathrm{mol}. \end{array}$$

One cannot but notice the fact that the double salt MgCl_2_·CsCl·6H_2_O formation from simple salts reaction (Equation (2)) has a positive thermal effect and the reaction occurs with the heat absorption. Most likely, this effect is the consequence of the observed changes between the surrounding of Cs in simple CsCl and double MgCl_2_·CsCl·6H_2_O salts. The break of CsCl lattice and the following formation of the double salt requires external heat. Moreover, it means the transformation of the cubic surrounding of Cs to an octahedral one, which is the major difference between structures of constituent and double salts. With the aid of the literature data [29] for MgCl_2_·CsCl·6H_2_O on the changes of the formation Gibbs energy from the simple salts $\Delta_{r}G_{298}^{0}=-8.668$ kJ/mol, it is possible to calculate the entropy of the reaction Equation (2) $\Delta_{r}S_{298}^{0}=345$ J·mol${}^{-1}$K${}^{-1}$. The entropy change for the reaction is high and the $T\;\cdot \;\Delta_{r}S_{298}^{0}>\Delta_{r}H_{298}^{0}$, whereas the $\Delta_{r}G_{298}^{0}$ is negative, but very small. The formation of the double salt from its constituents is impeded according to the thermodynamical functions:

(5) $$\Delta S=\frac{\Delta H-\Delta G}{T}$$

The dissolution heat of the double salt and of the mixture of the constituents salts was experimentally measured in order to calculate the $\Delta_{f}H_{298}^{0}$ of the double salt MgCl_2_·CsCl·6H_2_O to be −2847 kJ/mol. The reaction shown in Equation (2) is endothermic, and its enthalpy is evaluated to be equal to 94±4 kJ/mol.The heat of the reaction Equation (2) defines the reaction type as endothermic, and was calculated to be 94±4 kJ/mol. The entropy of the reaction was determined on the basis of the literature data to be $\Delta_{r}S_{298}^{0}=345$ J·mol${}^{-1}$K${}^{-1}$.

The results obtained lead us to the conclusion that the major factor in the formation of the joint compound from the constituents is the entropic factor. As it has been mentioned above (see the discussion section for the X-ray experimental results), the distances between polyhedra both for Mg and Cs in the structure of MgCl_2_·CsCl·6H_2_O are higher compared to simple salts. It leads us to the supposition that the energy of MgCl_2_·CsCl·6H_2_O crystal structure is lower than the sum of energies of CsCl and MgCl_2_·6H_2_O. Since the dissolution was carried out to infinite dilution, it can be assumed that the hydration energy for double salt and the mixture of components is the same. Then, the difference between the thermal effect of dissolving double salt and the sum of the thermal effect of dissolving simple salts should be endothermic. This is proved by our experiments.

### 3.3. Dilution Calorimetry

The molar differential dilution enthalpies were measured by the differential dilution calorimetry method for the solutions of the concentrations listed in Table 3. The data were used to investigate the behavior of partial molal entropy of water dependence on the concentration.

**Figure 6:**
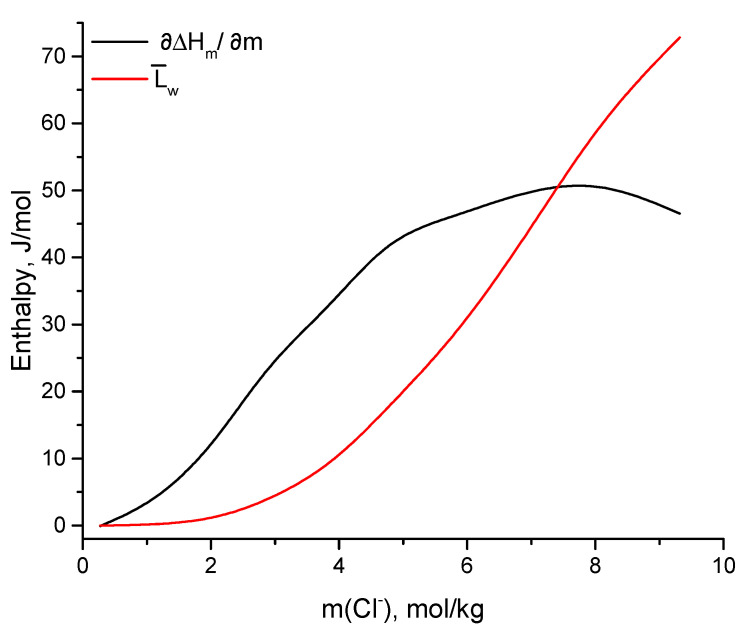
Partial molar ($\partial \Delta H_{m}/\partial m$) and relative partial molar (${\bar{L}}_{w}$) enthalpies dependency on total Cl^−^ molality.

In Table 3, the results of dilution calorimetry experiment for 10 ternary solutions are presented. Using Equation (6) ([30]), the relative partial molal enthalpies of the solvent ${\bar{L}}_{w}$ were calculated from experimentally obtained partial molal enthalpies. The results are shown in Table 3, and the comparison between these two enthalpies is presented in Figure 6.

(6) $$\Delta {\bar{L}}_{w}=\frac{m^{2}}{m_{w}}\;\cdot \;\frac{\partial \Delta H_{m}}{\partial m},$$

where *m* is total molality of the solution that can be calculated as $m=\sum_{i}m_{i}\;\cdot \;\nu_{i}$ (*i*= CsCl, MgCl_2_); $\nu_{i}$ is the total number of ions yielding from the dissociation of the salt. $m_{w}=55.508$ mol/kg is water molality. $\partial \Delta H_{m}/\partial m$ is experimentally obtained partial enthalpy of dilution [31].

The second step in the data analysis was to obtain the appropriate water activities for concentrations under study. From the literature data [13], the water activities for the solutions with $\begin{array}{c} {\mathrm{Cs}}^{+} \end{array}/\begin{array}{c} {\mathrm{Mg}}^{2+} \end{array}=1.8$ were acquired. After that, they were recalculated to water activities for the concentrations under study. The results are presented in Table 3. Retrieved activities were needed to calculate the chemical potential for each solution as given by the following equation:

(7) $$\Delta \mu_{wi}=RT\;\cdot \;ln\left(a_{wi}\right),$$

where $\Delta \mu_{wi}$ is the difference of water chemical potential in pure water solution and in *i* solution, *R* is a universal gas constant, and T=298.15 K.

The total relative partial molar entropy stands for the entropy change of the solution, when the infinitely small amount of the solvent is added to the infinitely large amount of solution. It was calculated as given below:

(8) $$\Delta {\bar{S}}_{w}^{t}=\frac{\Delta {\bar{L}}_{w}-\Delta \mu_{w}i}{T}.$$

The relative partial molar ideal entropy of the solvent (water) $\Delta {\bar{S}}_{w}^{i}$, which presumes the interaction among different molecules as the interaction among identical atoms and molecules, would depend on its mole fraction χ:

(9) $$\Delta {\bar{S}}_{w}^{i}=-R\;\cdot \;\ln\left(\chi \right).$$

The excess relative partial molar entropy describes the deviations of the real solution from the ideal solution. This entropy was found according to the following formula:

(10) $$\Delta {\bar{S}}_{w}^{e}=\Delta {\bar{S}}_{w}^{t}-\Delta {\bar{S}}_{w}^{i}.$$

Figure 6 shows the dependence of the partial molar ($\partial \Delta H_{m}/\partial m$) and relative partial molar (${\bar{L}}_{w}$) enthalpies for water solvent on the total Cl^-^ molality of the ternary system. As it can be seen, the dependence of the experimentally measured partial molar enthalpy steadily goes up approximately 8 mol/kg of total Cl^-^. This concentration corresponds to the eutectic point of the ternary system at the concentration ratio Cs^+^/Mg^2+^ =1.8 [32]. On the graph, we see the growths of the partial molar enthalpy in the beginning, which represents the endothermic effect, meaning the addition of small portion of water to the solution and the subsequent solvation of this water requires additional energy. At pre-eutectic concentration, there is a huge amount of free water molecules, compared to the water molecules solvating Mg^2+^, Cs^+^, Cl^−^ ions. The free water molecules are connected with each other by means of strong hydrogen-bonds. The introduction of new water to the solution makes hydrogen bonds break and reorients towards additional water molecules. The injection of small amount of the component (water) into the equilibrated system (solution) leads to the disturbance of the D-structure and heat absorbance. Furthermore, the rise of concentrations drives the D-structure of the solution further away from that of the introduced component, taking more energy for the equilibration process.

With further growth of concentration in the region of eutectic concentration, the slow deceleration of the endothermic effect trend is observed. It is known [33,34,35,36,37] that, at this point, the change of the structural dominant occurs. The water molecules can no longer be regarded as a solvent, but as a solute or as an inclusion into new dominant structure. In addition, the amount of free water molecules in comparison with water molecules solvating ions is very low. The calorimetric measurements, in particular, differential enthalpy of dilution, as it can be seen, are sensitive to the transition of the solution beyond eutectic concentration. Despite being mobile, water molecules have a different life-time in the nearest surroundings of different ions; as soon as Mg^2+^ is a kosmotropic ion, water molecules linger a considerable amount of time in its hydration shell and the hydration itself is exothermic reaction. At such high concentrations with the introduction of water molecules, the exothermic effect of the ions hydration overbalances the endothermic effect of the D-structure breakage.

In its turn, the relative partial molar enthalpy increases with growths of the concentration throughout all the measured concentration range. The ${\bar{L}}_{w}$ enthalpy describes the difference between the state under study and the reference state of the ideal solution (infinitely diluted solution). According to the graph, before m$\left(\begin{array}{c} {\mathrm{Cl}}^{-} \end{array}\right)$ = 2 mol/kg, the solution under study can be described with rules suitable for the infinitely diluted solutions because the change of ${\bar{L}}_{w}$ is very small in this region. Onward, after this concentration, relative partial molar enthalpy experiences a dramatic increase and reflects the state of the water molecules in studied solutions. One can no longer call them ideal, and it is necessary to take the ion–ion interactions into consideration.

**Figure 7:**
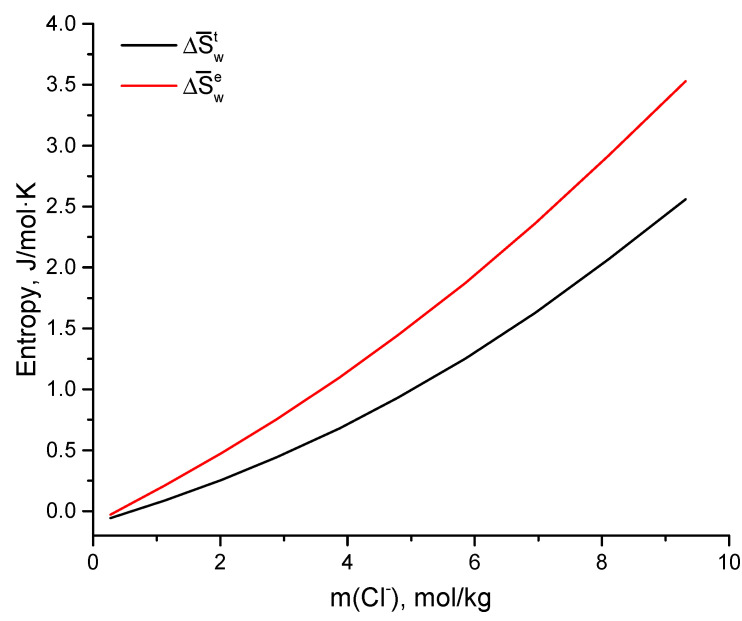
Dependence of total relative partial molar $\Delta {\bar{S}}_{w}^{t}$ and excess relative partial molar $\Delta {\bar{S}}_{w}^{e}$ entropies on total Cl^−^ molality.

The dependence of relative partial molar $\Delta {\bar{S}}_{w}^{t}$ and excess relative partial molar $\Delta {\bar{S}}_{w}^{e}$ entropies on total Cl^−^ concentration are presented in Figure 7. The growths of the $\Delta {\bar{S}}_{w}^{t}$ depend on two factors.

The first one is the mobility increase for the fraction of the water molecules. Insofar as the water molecules are leaving the third and second hydration shells of ions, with a further increase of concentration and formation of solvent-separated and then contact ion pairs. This is facilitated by the chaotropic nature of the cesium ion [38]. Additionally, the amount of CsCl in the solution is twice as much as magnesium chloride. At the same time, Mg^2+^ as a kosmotropic ion inclines to form the first solvation shell with six water molecules, and has an ability to form the second solvation shell with water molecules even in concentrated solutions [39].

The second factor defining the increase of entropy is thermodynamic probability of the system, the appearance of many different structures containing water. In solution, the diversity of structures containing water rises with growths of concentration, which influences the trend of entropy.

## 4. Conclusions

In this paper, the novel studies of the liquid and solid phases pertaining to the ternary MgCl_2_−CsCl−H_2_O system are presented. The analysis of the structure of solid compounds (MgCl_2_·6H_2_O·CsCl, CsCl, MgCl_2_·6H_2_O) revealed the loosening of the double salt structure in comparison with simple salts, such as the dramatic increase of distances between corresponding atoms and the separation of the tightly connected Cs polyhedra. According to the dissolution calorimetry results, the loosening was reflected in the endothermic enthalpy for double salt formation from simple salts, insofar as the crystal structure energy of the double salt MgCl_2_·6H_2_O·CsCl is lower than the sum of those for simple constituent MgCl_2_·6H_2_O and CsCl salts. The standard formation enthalpy $\Delta_{f}H_{298}^{0}$ of the double salt MgCl_2_·CsCl·6H_2_O was calculated to be −2847±4 kJ/mol by use of the dissolution calorimetry method. The reaction heat of the double salt formation from simple salts was defined to be 94±4 kJ/mol. The entropy of the reaction was determined with the help of the literature data to be $\Delta_{r}S_{298}^{0}=345$ J/mol${}^{-1}$ K ${}^{-1}$. The molar differential dilution enthalpy is endothermic throughout all the concentration range according to the dilution calorimetric experiment results. The combination of the conclusions from calorimteric measurements and X-ray experimental data shows that the formation of double salt from simple salts as well as the formation of joint compound from ternary solution is due to the increase of the entropy factor. The significant value of dissolution enthalpy of double salt makes it attractive for use as a thermochemical energy storage.

## Figures and Tables

**Table 1:** The comparison of distances between neighboring atoms for simple CsCl and double salt.

Salt	d(Cs-Cs), Å	d(Cs-Cl), Å
MgCl_2_·CsCl·6H_2_O	6.7275	3.3686
CsCl	4.1150	3.5637

**Table 2:** The comparison of distances between neighboring atoms for simple MgCl_2_·6H_2_O and double salt.

Salt	d(Mg-Mg), Å	d(Mg-O), Å
MgCl_2_·CsCl·6H_2_O	6.7258	2.051–2.071
MgCl_2_·6H_2_O	6.0737	2.0573–2.0620

**Table 3:** The parameters obtained from dilution calorimetry experiment.

MgCl_2_	CsCl	Cl^−^	$a_{w}$	$\partial \Delta H_{m}/\partial m$	$\Delta {\bar{L}}_{w}$	$\Delta {\bar{S}}_{w}^{i}$	$\Delta {\bar{S}}_{w}^{e}$	$\Delta {\bar{S}}_{w}^{t}$
mol/kg					J/mol		J/mol·K		
2.45	4.41	9.32	0.67	46.55	72.81	0.97	2.56	3.53
2.13	3.84	8.11	0.72	51.47	60.92	0.85	2.07	2.92
1.83	3.29	6.95	0.77	50.13	43.63	0.73	1.63	2.36
1.54	2.77	5.85	0.81	46.30	28.57	0.62	1.25	1.87
1.26	2.27	4.79	0.85	43.23	17.88	0.51	0.93	1.44
1.02	1.84	3.88	0.88	32.83	8.89	0.42	0.68	1.10
0.76	1.37	2.88	0.91	24.14	3.62	0.31	0.44	0.75
0.53	0.96	2.03	0.94	11.59	0.86	0.22	0.26	0.48
0.30	0.54	1.13	0.97	3.50	0.08	0.12	0.09	0.21
0.07	0.13	0.27	1.00	−0.10	0.00	0.03	−0.06	−0.03

## Data Availability

Results data can be obtained upon request to the corresponding author.

## Abbreviations

The following abbreviations are used in this manuscript:

XRC	X-ray crystallography
$\Delta H_{sol}$	The dissolution enthalpy
$\Delta_{f}H_{298}^{0}$	The standard formation enthalpy
$\Delta_{r}G_{298}^{0}$	The Gibbs energy change of the reaction
$\Delta_{r}S_{298}^{0}$	The entropy change of the reaction
$\Delta_{r}H_{298}^{0}$	The enthalpy change of the reaction
$\Delta {\bar{L}}_{w}$	The relative partial molal enthalpy of the solvent (water)
$\partial \Delta H_{m}/\partial m$	The molar differential dilution enthalpy
$\Delta {\bar{S}}_{w}^{t}$	The total relative partial molar entropy
$\Delta {\bar{S}}_{w}^{i}$	The ideal relative partial molar entropy
$\Delta {\bar{S}}_{w}^{e}$	The excess relative partial molar entropy

## Appendix A

**Table A1:** Details of the calorimeter calibration with KCl.

Mass, g	ν, mol	S	|Q|, kJ	K	Error
0.0273	3.66 × 10${}^{-4}$	1.644	6.32	0.260	0.008
0.0232	3.11 × 10${}^{-4}$	1.468	5.37	0.274	−0.005
0.0232	3.11 × 10${}^{-4}$	1.408	5.37	0.262	0.006
0.0282	3.78 × 10${}^{-4}$	1.652	6.53	0.253	0.015
0.0315	4.23 × 10${}^{-4}$	1.949	7.29	0.267	0.001
0.0218	2.92 × 10${}^{-4}$	1.428	5.04	0.283	−0.015
0.0244	3.27 × 10${}^{-4}$	1.559	5.65	0.276	−0.008
0.0203	2.72 × 10${}^{-4}$	1.266	4.70	0.270	−0.001
				0.268	±0.021

where *m* is the mass of the sample, *v* is the mole of the sample, *S* is an integral of the curve, |Q| is a module of the heat effect calculated as *Q* = *v*·*Δ*H_*sol*_(*KCl*)_*s*_·1000, and *K* is a calorimeter constant *K* = *S*/*Q*.

**Table A2:** Details of the $\Delta H_{sol}\left(\begin{array}{c} {\mathrm{MgCl}}_{2}\cdot \mathrm{CsCl}\cdot 6H_{2}O \end{array}\right)$ measurements.

Mass, g	ν, mol	S	|Q|, kJ	$\Delta H_{\mathrm{sol}}$, kJ	Error
0.0235	6.32 × 10${}^{-5}$	1.905	7.11	−112	1.79
0.0370	9.95 × 10${}^{-5}$	2.928	10.93	−110	−0.88
0.0302	8.12 × 10${}^{-5}$	2.350	8.77	−108	−2.71
0.0392	1.05 × 10${}^{-5}$	3.678	11.86	−112	1.80
				−128	±4.07

**Table A3:** Details of the $\Delta H_{sol}\left(\begin{array}{c} {\mathrm{MgCl}}_{2}\cdot 6H_{2}O \end{array}+\begin{array}{c} \mathrm{CsCl} \end{array}\right)$ measurements.

m(MgCl_2_·6H_2_O), g	m(CsCl), g	S	|Q|, kJ	$\Delta H_{\mathrm{sol}}$, kJ	Error
0.0264	0.0224	1.179	4.04	−16.73	−0.52
0.0275	0.0259	1.355	5.06	−17.49	0.24
0.0135	0.0119	0.626	2.34	−17.05	−0.20
0.0135	0.0112	0.622	2.32	−17.45	0.20
				−17.25	±0.66

**Table A4:** X-ray diffraction parameters of double salt MgCl_2_·CsCl·6H_2_O.

Parameter	Value
Formula sum	Cl3 H12 Cs Mg O6
Formula weight	371.69 g/mol
Crystal system	monoclinic
Space-group	C 1 2/c 1 (15)
Cell parameters	a = 9.4134(3) Å b = 9.6425(3) Å c = 13.4708(3) Å $\beta =90.280{\left(3\right)}^{\circ }$
Cell ratio	a/b = 0.9762 b/c = 0.7158 c/a = 1.4310
Cell volume	1222.71(6) Å${}^{3}$
Z	4
Calc. density	2.01891 g/cm${}^{3}$
RAll	0.0322
Pearson code	mC100
Formula type	NO2P4Q6R12
Wyckoff sequence	f12e

## References

[1] Høy-Petersen N., Rizley J.H. (2020). Magnesium Processing.

[2] Tavares J., Moura L., Bernardo A., Giulietti M. (2018). Crystallization and separation of KCl from carnallite ore: Process development, simulation, and economic feasibility. Chem. Ind. Chem. Eng. Q..

[3] Prokhorov A., Knunyants I. (1988). Khimicheskaia Entsiklopedia: V Piati Tomakh (Chemical Encyclopedia in 5 Volumes).

[4] Mamani V., Gutiérrez A., Fernández A., Ushak S. (2020). Industrial carnallite-waste for thermochemical energy storage application. Appl. Energy.

[5] Gutierrez A., Ushak S., Mamani V., Vargas P., Barreneche C., Cabeza L.F., Grágeda M. (2017). Characterization of wastes based on inorganic double salt hydrates as potential thermal energy storage materials. Sol. Energy Mater. Sol. Cells.

[6] Gutierrez A., Ushak S., Linder M. (2018). High Carnallite-Bearing Material for Thermochemical Energy Storage: Thermophysical Characterization. ACS Sustain. Chem. Eng..

[7] Emons H.H. (1988). Mechanism and kinetics of formation and decomposition of carnallitic double salts. J. Therm. Anal..

[8] Emons H., Brand P., Pohl T., Köhnke K. (1988). Crystal chemistry of MX·MgX_2_·6H_2_O type compounds. ZAAC J. Inorg. Gen. Chem..

[9] Emons H.H., Naumann R., Pohl T., Voigt H. (1984). Thermoanalytical investigations on the decomposition of double salts. J. Therm. Anal..

[10] Balarew C., Tepavitcharova S. (1990). Co-Crystallization in systems with carnallite-type double salts. Z. Für Anorg. Und Allg. Chem..

[11] Hu M., Zhang W., Li S., Zhai Q., Jiang Y. (2009). Thermodynamic investigation of a ternary mixed electrolyte (CsCl/MgCl_2_/H_2_O) system using electromotive force measurement at 298.15 K. J. Chem. Eng. Data.

[12] Guo L., Tu L., Wang Y., Li J. (2018). Water Activity and Solubility Measurements and Model Simulation of the CsCl−MgCl_2_−H_2_O Ternary System at 323.15 K. J. Chem. Eng. Data.

[13] Guo L., Wang Y., Tu L., Li J. (2017). Thermodynamics and Phase Equilibrium of the System CsCl−MgCl_2_−H_2_O at 298.15 K. J. Chem. Eng. Data.

[14] Skoog D.A., West D.M., Holler F.J. (1996). Fundamentals of Analytical Chemistry.

[15] Pestova O., Bukesova V., Kondrat’ev Y., Khripun V., Baranauskaite V. (2018). Energy Characteristics of Lithium–Cesium Binary Chloride Dissolution in Water. Russ. J. Gen. Chem..

[16] Kilday M. (1980). The enthalpy of solution of SRM 1655 (KCl) in H_2_O. J. Res. Natl. Bur. Stand..

[17] Waizumi K., Masuda H., Ohtaki H., Scripkin M.Y., Burkov K.A. (1991). Crystallographic investigations of [Mg(H_2_O)_6_]XCl_3_ double salts (X^+^ = K^+^, Rb^+^, Cs^+^, NH4^+^): Crystal structure of [Mg(H_2_O)_6_]CsCl_3_. Am. Mineral..

[18] Ahtee M. (1969). Lattice constants of some binary alkali halide solid solutions. Ann. Acad. Sci. Fenn. Ser. A Phys..

[19] Agron P.A., Busing W.R. (1985). Magnesium dichloride hexahydrate, MgCl_2_·6H_2_O, by neutron diffraction. Acta Crystallogr. Sect. C Cryst. Struct. Commun..

[20] Andress K.R., Carpenter C. (1934). Kristallhydrate. Z. Krist. Cryst. Mater..

[21] Khripun M., Karavan S., Bulgakov S. (1987). Vzaimosvjaz’ struktury i stroenija v koncentrirovannyh rastvorah jelektrolitov. (The correlation of the structure and the order in the concentrated electrolyte solutions). Probl. Sovrem. Him. Koord. Soedin. Quest. Contemp. Chem. Complex Compd..

[22] Naumov G.B., Ryzhenko B.N., Khodakovski I.L., Barnes I., Speltz V. (1974). Handbook of Thermodynamic Data.

[23] (1997). Fiziko-Chimiceskie Svojstva Galurgiceskich Rastvorov i Solej: Chloridy Natrija, Kalija i Magnija; Spravocnik.

[24] Waizumi K., Masuda H., Ohtaki H., Burkov K.A., Scripkin M.Y. (1991). Structure of MgCl_2_·RbCl·6H_2_O. Acta Crystallogr. Sect. C Cryst. Struct. Commun..

[25] Travis W., Glover E.N.K., Bronstein H., Scanlon D.O., Palgrave R.G. (2016). On the application of the tolerance factor to inorganic and hybrid halide perovskites: A revised system. Chem. Sci..

[26] Shomate C.H., Huffman E.H. (1943). Heats of Formation of MgO, MgCl_2_, MgCl_2_·H_2_O, MgCl_2_·2H_2_O, MgCl_2_·4H_2_O, and MgCl_2_·6H_2_O. J. Am. Chem. Soc..

[27] Johnson G.K., Gayer K.H. (1979). The enthalpies of solution and formation of the chlorides of cesium and rubidium. J. Chem. Thermodyn..

[28] Li D., Zeng D., Yin X., Gao D., Fan Y. (2020). Phase diagrams and thermochemical modeling of salt lake brine systems. IV. Thermodynamic framework and program implementation for multicomponent systems. Calphad.

[29] Balarew C., Christov C., Valyashko V., Petrenko S. (1993). Thermodynamics of formation of carnallite type double salts. J. Solut. Chem..

[30] Pepple G.W. (1967). Relative Apparent Molal Heat Contents of Some Aqueous Rare-Earth Chloride Solutions at 25  C. Ph.D. Thesis.

[31] Mishchenko K.P., Poltoratskiy G.M. (1976). Termodinamika i Stroenie Vodnyh i Nevodnyh Rastvorov Elektrolitov (Thermodynamics and Structure of the Water and Non-Water Electrolyte Solutions).

[32] Belysheva M., Pestova O., Baranauskaite V., Anufrikov Y. Dissolution and Dilution Enthalpies of the Ternary System MgCl2−CsCl−H2O at 298,15 K. Proceedings of the Mendeleev 2019 Conference.

[33] Enderby J.E., Neilson G.W. (1981). The structure of electrolyte solutions. Rep. Prog. Phys..

[34] Copestake A.P., Neilson G.W., Enderby J.E. (1985). The structure of a highly concentrated aqueous solution of lithium chloride. J. Phys. C Solid State Phys..

[35] Podolsky R.J. (1960). The Structure of Water and Electrolyte Solutions. Circulation.

[36] Efimov A.Y., Khripun M.K., Myund L.A., Pestova O.N. (2016). Mobile nanostructures (cybotactic groups) as a basis of generalised phenomenological model of aqueous electrolyte solutions. Int. J. Nanotechnol..

[37] Pestova O.N., Efimov A.Y., Myund L.A., Kudrev A.G., Khripun V.D., Davidian A.G., Baranauskaite V.E. (2017). Structural Inhomogeneity in Electrolyte Solutions: The Calcium Perchlorate–Water System. J. Solut. Chem..

[38] Pham V.T., Fulton J.L. (2018). Contact ion-pair structure in concentrated cesium chloride aqueous solutions: An extended X-ray absorption fine structure study. J. Electron Spectrosc. Relat. Phenom..

[39] Afanas’ev V.N., Ustinov A.N. (2012). Hydration numbers and the state of water in hydration spheres of magnesium chloride and magnesium sulfate solutions. Russ. J. Inorg. Chem..

